# Floral organ transcriptome in *Camellia sasanqua* provided insight into stamen petaloid

**DOI:** 10.1186/s12870-022-03860-x

**Published:** 2022-10-05

**Authors:** Menglong Fan, Xinlei Li, Ying Zhang, Si Wu, Zhixin Song, Hengfu Yin, Weixin Liu, Zhengqi Fan, Jiyuan Li

**Affiliations:** 1 Research Institute of Subtropical Forestry, Chinese Academy of Forestry, Hangzhou, 311400 Zhejiang China; 2 Research Institute of Forestry, Chinese Academy of Forestry, Beijing, 100091 China

**Keywords:** *Camellia sasanqua*, Double flower, Transcriptome, Petaloid stamen, ABCE model, Phytohormone

## Abstract

**Background:**

The cultivated *Camellia sasanqua* forms a divergent double flower pattern, and the stamen petaloid is a vital factor in the phenomenon. However, the regulation mechanism remains largely unclear.

**Results:**

Here, a comprehensive comparative transcriptome analysis of the wild-type, “semi-double”, “peony double”, and “rose double” was performed. The cluster analysis of global gene expression level showed petal and stamen difficulty separable in double flower. The crucial pathway and genes related to double flower patterns regulation were identified by pairwise comparisons and weighted gene coexpression network (WGCNA). Divergent genes expression, such as *AUX1* and *AHP*, are involved in plant hormone signaling and photosynthesis, and secondary metabolites play an important role. Notably, the diversity of a petal-specific model exhibits a similar molecular signature to the stamen, containing extensin protein and *PSBO1*, supporting the stamen petaloid point. Moreover, the expansion of class A gene activity influenced the double flower formation, showing that the key function of gene expression was probably demolished.

**Conclusions:**

Overall, this work confirmed the ABCE model and provided new insights for elucidating the molecular signature of double formation.

**Supplementary Information:**

The online version contains supplementary material available at 10.1186/s12870-022-03860-x.

## Background

*Camellia* (family Theaceae) contains about 250 species [1], *C. japonica* (ornamental), *C. sinensis* (beverage), *C. oleifera* (oil). *Camellia sasanqua* belongs to section *oleifera*, mainly in tropical and sub-tropical zones. Nevertheless, the research on *C. sasanqua* mainly focused on the flowers’ pigmentation [2, 3], little is known about its flower pattern, especially the development of the stamen petaloid. There are four whorls in its flower, some sepals in the first, five petals in the second, numerous stamens in the third, and one carpel in the fourth. Interestingly, some flower patterns are mutated in cultivated cultivars, including semi-double, peony double, rose double, and anemone double (according to the criteria set out by the international camellia society). In these heavily petaled flowers, the stamens become, to varying degrees, petal-like organs, somehow representing the mitigated stamen growth in camellia [4]. Despite extensive knowledge of the molecular regulation mechanism of a flower pattern change in model plants, it remains unknown how the floral pattern in *C. sasanqua* cultivars is achieved.

Most floral organs contain four parts, petal, stamen, sepal, and carpel, and their development is influenced by conserved molecular mechanisms [5]. In the model plant, the ABCE model relates to flower development [6, 7]. Class-A (*APETALA1*, *APLETALA2*, *LIPLESS1*, and *LIPLESS2*) and class-E genes (*SEPALLATA*) control the development of sepals. Class-A, class-B (*APLETALA3*, *PISTILLATA*, *DEFICIENS*, and *GLOBOSA*), and class-E genes regulate the characteristics of petals. Class-B, class-C (*AGAMOUS*, *PLENA*, and *FARINELLI*), and class-E genes determine the stamen phenotype [8]. The previous study showed the A, 2B, and E tetramers regulate the formation of petals [9]. In addition, phytohormones also play a primary role in flower change [10], and photosynthesis provides nutrition for reproductive development [11, 12], floral diversification promotes reproductive success through interaction with pollinators [6]. Overall, a complicated genetic pathway network control flower architecture. Although the tenets are conserved in angiosperms, different families show different characteristics, such as hundreds of independent carpels arranged on the receptacle in *Fragaria* × *ananassa* [13], and stamen petaloid in *Alcea rosea* [14]. Abundant information is required for understanding the variation of double flowers.

The present study generated comparative floral organs transcriptome data of wild-type and three double-flower cultivated *C. sasanqua* by taking advantage of the Illumina platform. As a result, transcription change related to double-flower formation was captured, and tissue-specific gene modules were identified by WGCNA. Most ABCE homeotic genes were expressed in expected floral organs. Together, the gene expression profile described here provides the foundation for molecular signature exploration of the *C. sasanqua* flower pattern.

## Results

### Phenotype divergence among four kinds of flower pattern

**Fig. 1:**
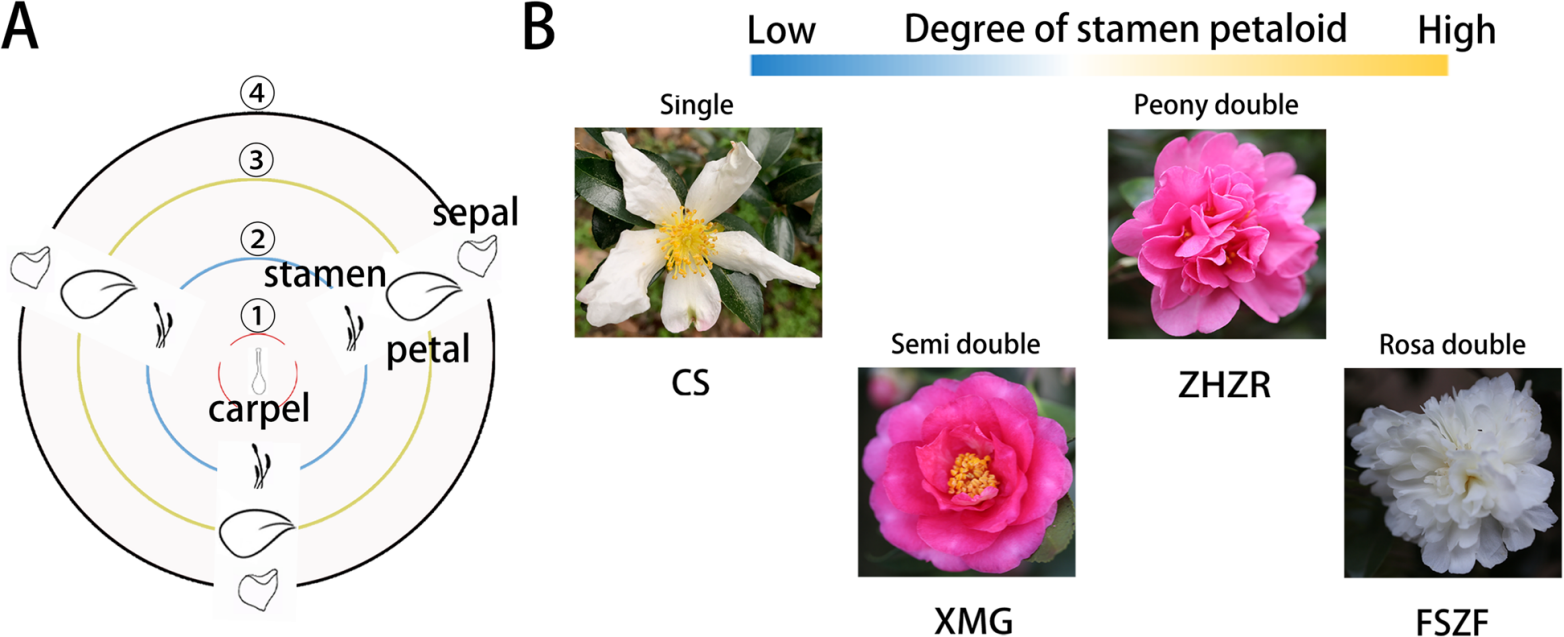
Phenotype divergence of *C. sasanqua* wild-type (single flower) and cultivated double flowers. **A** The diagram illustrates the four whorls of the floral organ in *C. sasanqua*, including carpel, stamen, petal, and sepal. **B** Whole flower comparison among single flower, semi-double, rose double, and peony double

The composition of floral organs influences flower patterns, and further improves ornamental value and reproductive capacity. In general, the flower of *C. sasanqua* contains carpels, stamens, petals, and sepals (Fig. 1A). With the increase of the degree of stamen petaloid, the number of stamens decreased, and the number of petals increased, forming many double-flower variants (Fig. 1B), such as semi-double (XMG), peony double (ZHZR), and rose double (FSZF). By analyzing the transcriptome divergence among flower tissues, we can further reveal the molecular characteristic of stamen petaloid in *C. sasanqua*.

### General description of transcriptome data

The quality of 36 RNAs sequencing data collected from sepal, stamen, and petal of *C. sasanqua* with different flower patterns are listed in Supplemental Table S1. The number of clean reads per library ranged from 22 to 41 million, and the average CleanQ30 > 93%. The mapping rate to the reference genome [15] ranged from 75.25% to 82.63%, and more than 75.7% of the reads were mapped to the exon region (Supplemental Table S1). The high-quality data were used to perform further analysis. A total of 42, 463 genes were identified and qualified based on the Fragments Per Kilobase Million (FPKM) values. The correlation analysis showed similar expression patterns for all the biological replicates (Supplemental Fig. S1A).

Cluster analysis of the organs' global expression levels showed that 36 samples were divided into two clusters, petal and stamen formed one group, and sepal formed a distinct section (Supplemental Fig. S1B). In the CS, each floral organ exhibits distinct morphology and is easily separable. However, petals and stamens are difficult to separate in double flowers, providing evidence of a stamen petaloid at the transcriptional level.

### Pairwise differential expression observation of floral tissue

**Fig. 2:**
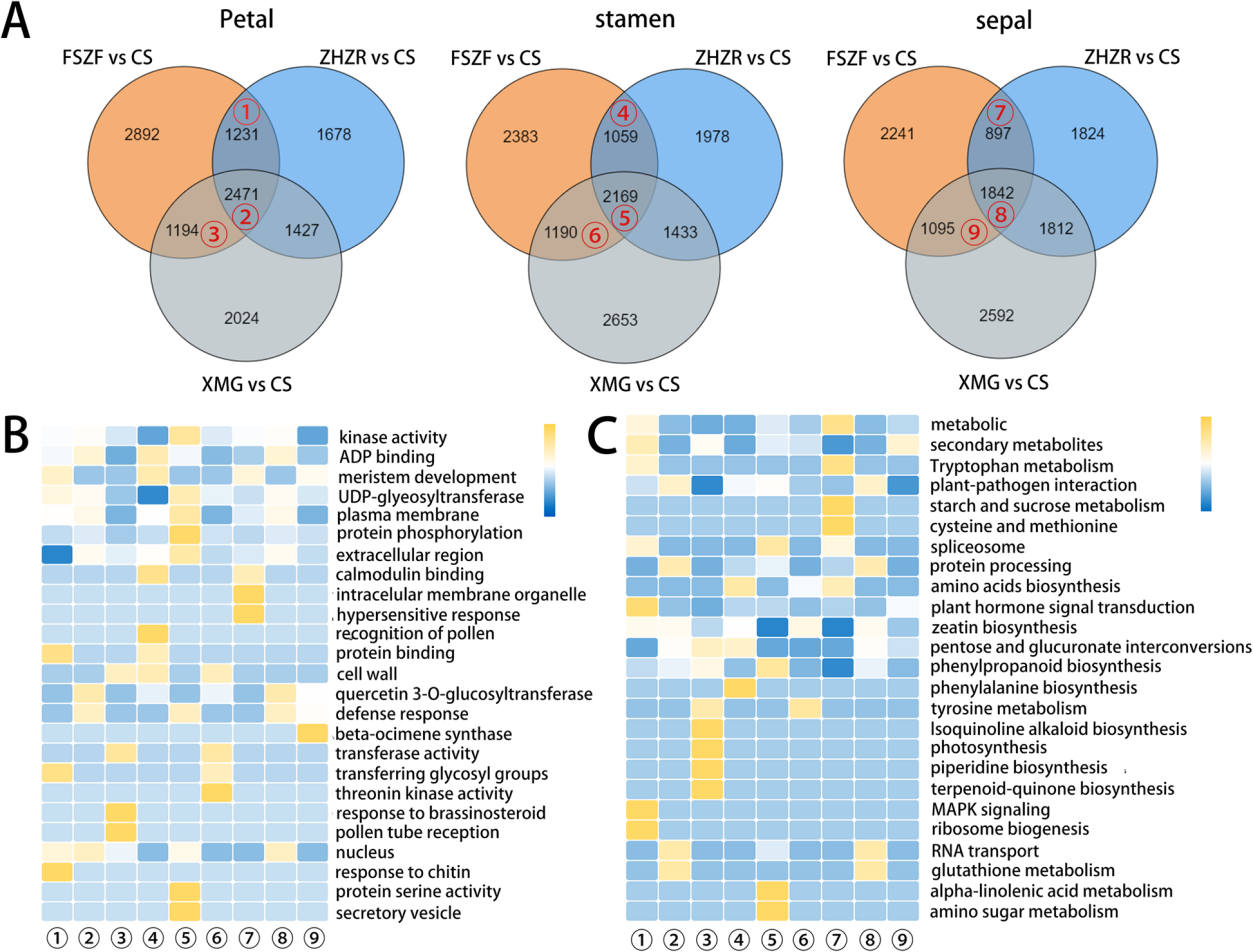
The transcription divergence of the floral organ. **A** Venn plots of DEGs among homologous organs of different flower patterns, the red mark indicates a specified comparison group. **B** The highly enriched GO terms and their distributions among comparisons. The number indicate the overlap mark in (**A**). **C** The highly enriched KEGG pathway and their distributions among comparisons. The numbers indicate the overlap mark in A

To investigate the transcription divergence that formed different flower patterns, strict screening criteria (|log2FC|≥ 1 and FDR < 0.05) were used. In a comparative analysis of homologous organs, the petal, stamen, and sepal, shared 2471, 2169, and 1842 DEGs, respectively (Fig. 2A). The maximum number of DEGs (2892) was specific to the FSZF vs. CS comparison. Due to the interference of color in the XMG and ZHZR, FSZF vs. CS did not have an influence on color. So, we focused on the overlapping DEGs, such as 1194 were shared by XMG vs CS and FSZF vs CS comparison, and 1231 DEGs were shared by ZHZR vs CS and FSZF vs CS comparison.

Accordingly, the gene ontology (GO) enrichment analyses of the overlapping DEGs were performed and combined as a matrix keeping the significant GO terms (Fig. 2B and Supplemental Table S2). Significant enrichment was observed in the GO terms related to “kinase activity”, “meristem development”, “protein phosphorylation”, “cell wall”, and “response to brassinosteroid”. In addition, the Kyoto Encyclopedia of Genes and Genomes (KEGG) pathway enrichment result revealed enrichment of genes involved in the biosynthesis of secondary metabolites, plant hormone signal transduction, photosynthesis, and tryptophan metabolism (Fig. 2C, and Supplemental Table S3).

### Identification of tissue specific coexpression models

**Fig. 3:**
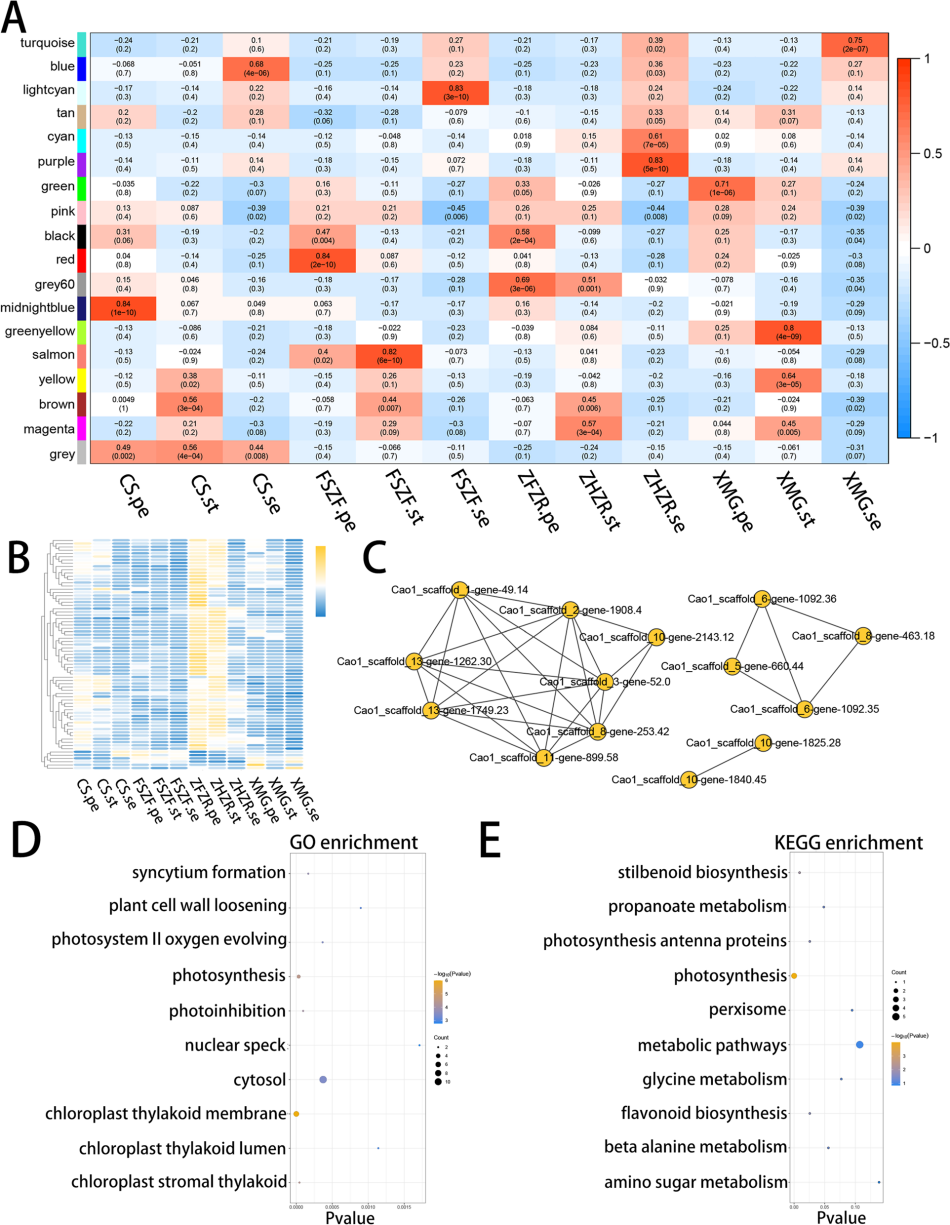
Weighted gene coexpression network analysis of floral organ. **A** Correlation between the gene model and the petal, stamen, and sepal of different flower patterns in the coexpression network. The correlation coefficient and the *p*-value are shown within each cell. The right panel is a color scale for correlating module traits from -1 to 1. **B** The expression heatmap of all genes in model grey60. Yellow and blue indicate high and low expression levels, respectively. **C** The correlation network of the grey60 module with the high edge weight as visualized by Cytoscape. **D** The top 10 enriched GO terms in the grey60 model. **E** The top 10 enriched KEGG pathways in the grey60 model

A total of 4, 247 genes with 10% of the variance were used for the weighted gene coexpression network analysis (WGCNA). A power 12 with a scale-free topological fit index of 0.9 was chosen, and 17 different models were obtained (showed in a different color). The model eigengene is the first principal component of a given module and can be considered a representative of the module’s gene expression profile. Twelve of these models correlated with a specific tissue, such as, blue (*r* = 0.68, *p* = 4e—6) and purple (*r* = 0.83, *p* = 5e—10) model identified sepal specific genes of CS and ZHZR, respectively (Fig. 3A). Interestingly, both petal and stamen of ZHZR were correlated with the grey60 model (Fig. 3B), indicating that molecular similarities between the petal and stamen in ZHZR may contain regulated genes of stamen petaloid. The development genes photosystem II oxygen-evolving enhancer protein 1 (Cao1_scaffold_10-gene-1860.33), extensin family protein (Cao1_scaffold_10-gene-2143.12), and gamma tonoplast intrinsic protein (Cao1_scaffold_13-gene-1017.19) (Supplemental Table S4) were observed in the grey60 model. In addition, a high-weight network by calculating the connectivity between gene modules was constructed and is shown in Fig. 3C.

Focusing on the grey60 model, GO and KEGG enrichment analyses were performed. Results showed significant enrichment in the GO terms related to photosynthesis and chloroplast (Fig. 3D and Supplemental Table S5), including “photosystem IIoxygen evolving complex”, “photoinhibition”, “chloroplast stromal thylakoid”. Moreover, “plant-type cell wall loosening” was also enriched, indicating that the cell process played an important role in double flower development. The first 10 KEGG pathways are involved in photosynthesis, flavonoid biosynthesis, and metabolic pathways (Fig. 3E and Supplemental Table S6).

### Phytohormone signal pathway involved in double flower development

**Fig. 4:**
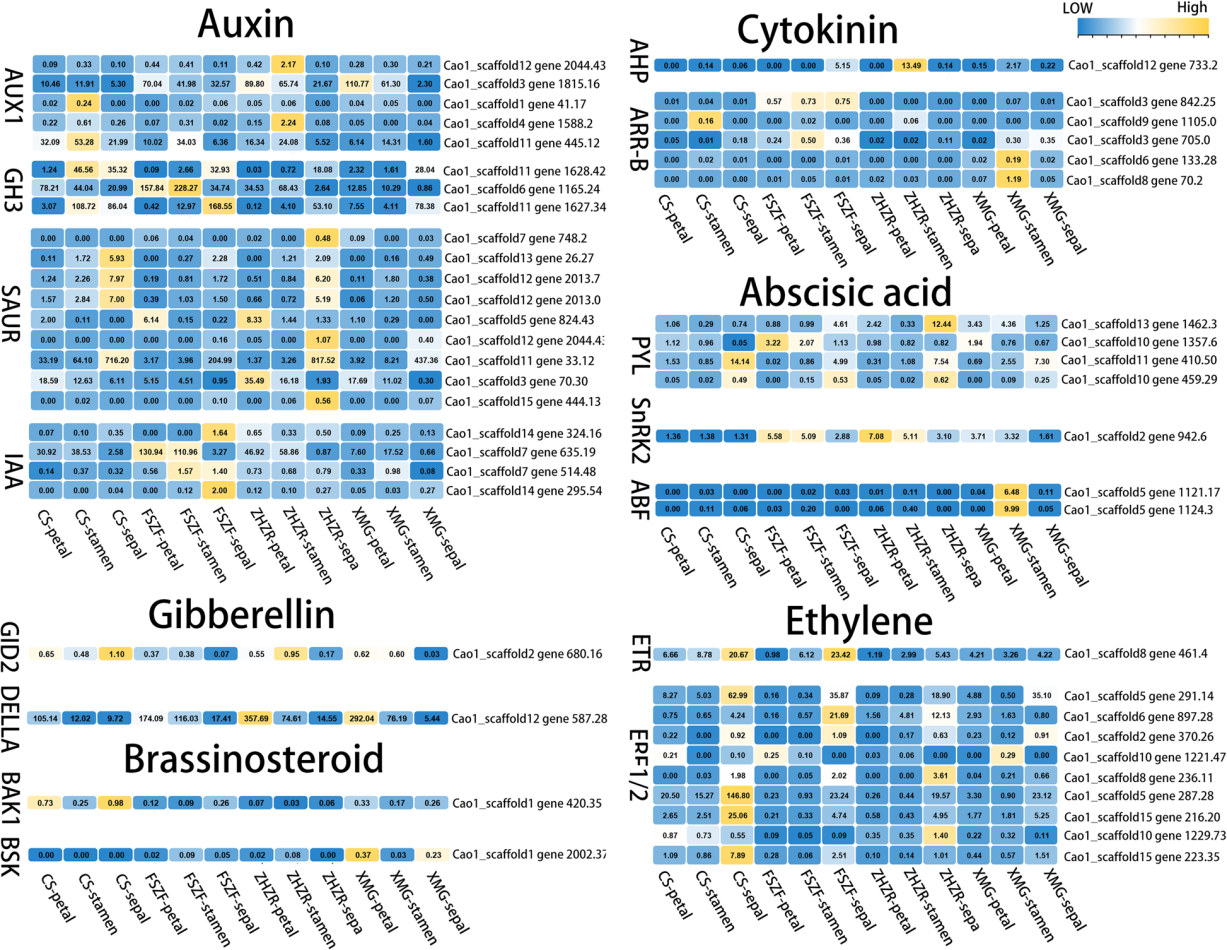
Expression heatmap of the DEGs involved in plant hormone pathways. RNA-seq data were normalized based on the mean expression value of each gene, yellow and blue indicate high and low expression levels, respectively

The above pathway analysis of overlapping DEGs revealed that plant hormones participate in double flower formation. Fifty-one DEGs were identified as regulating plant hormone signals (Fig. 4). Most genes were involved in auxin biosynthesis and signaling, five *AUX1*, four *IAA*, three *GH3*, and ten *SAUR* coding genes. *AUX1*, *IAA*, and *GH3* coding genes are upregulated in the double flower, particularly in petal and stamen. Interestingly, the *SAUR* coding genes have a high expression level in sepal. Moreover, *DELLA* (Cao1_scaffold_12-gene-587.28) in the Gibberellin pathway, *BSK* (Cao1_scaffold_1-gene-2002.37) in Brassinosteroid, *AHP* (Cao1_scaffold_12-gene-733.2) in Cytokinin, *SnRK2* (Cao1_scaffold_2-gene-942.6) in Abscisic acid were upregulated in double flower. However, *ERF1/2* (Cao1_scaffold_5-gene-287.28) coding gene was downregulated in double flower.

### ABCE homologous genes in *C. sasanqua*

**Fig. 5:**
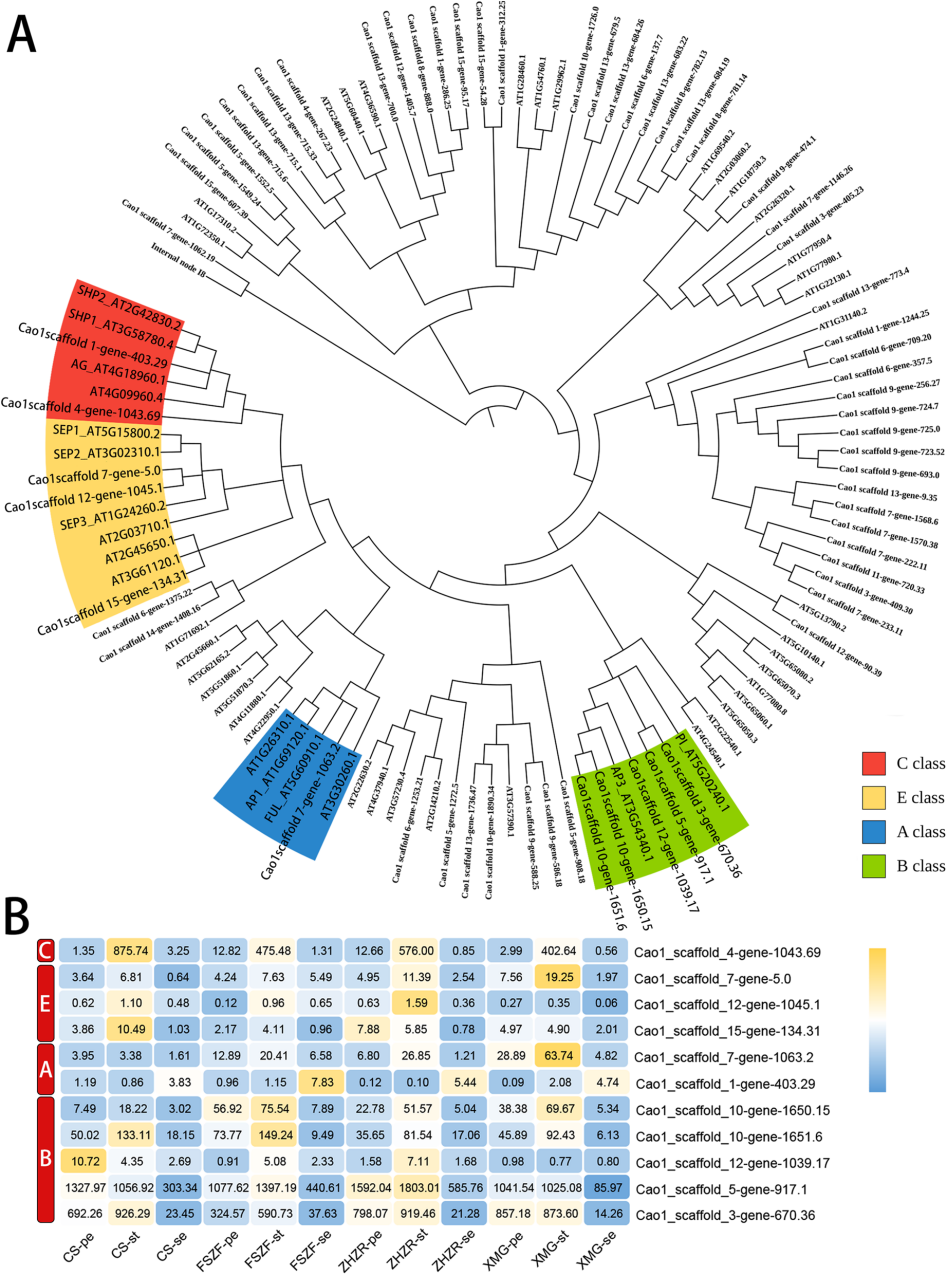
Identification and expression analysis of *MADS-box* genes. **A** Phylogenetic tree of *MADS-box* genes in *C. sasanqua* and *Arabidopsis*. The colored region indicated the ABCE model genes subgroups. **B** The expression heatmap of ABCE class genes in *C. sasanqua*, yellow and blue, indicate high and low expression levels, respectively

It is well known that floral structural variation is usually determined by homeotic genes in the ABCE model. To gain insight into transcription change in double flower development, we identified the *MADS-box* gene family regulating flower patterns. Synthesizing the results of Hmmsearch and Blast + method, a total of 65 sequences containing MADS-box and K-box domains were identified. These candidate genes were aligned with the MADS-box protein of *Arabidopsis thaliana*. For constructing a phylogeny tree (Fig. 5A). Finally, 11 homologs genes of A, B, C, and E classes were identified in our database.

**Fig. 6:**
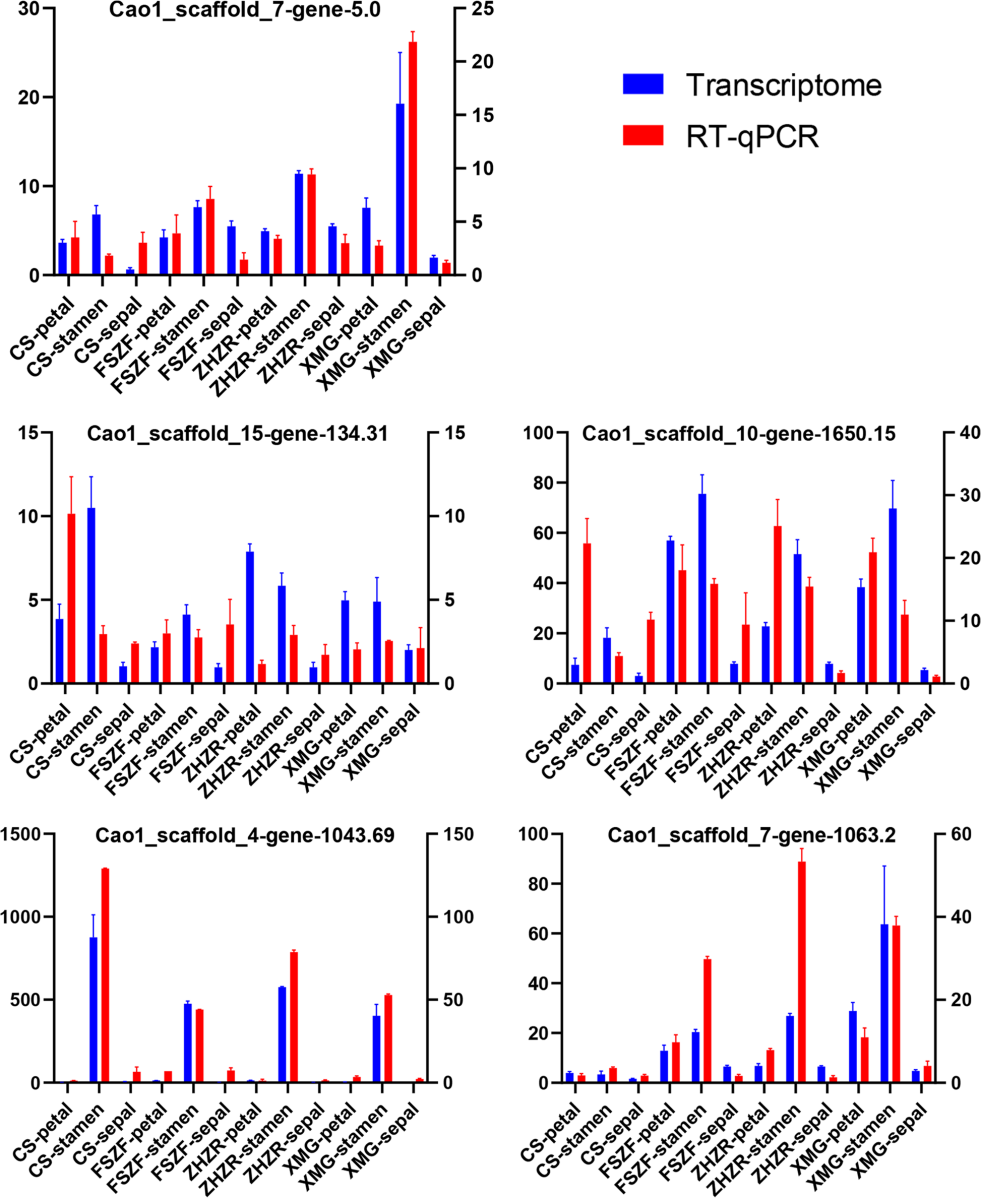
The expression levels of 5 genes at different tissues for RT-qPCR and the RNA-Seq experiment, red and blue indicated RT-qPCR and transcriptome data, respectively

The expression analysis showed that E class genes (Cao1_scaffold_7-gene-5.0, Cao1_scaffold_15-gene-134.31) were upregulated in double flower cultivars (Fig. 5B). One C class gene (Cao1_scaffold_4-gene-1043.69) mainly accumulated in stamen and was downregulated in the double flower. Two B class genes activity (Cao1_scaffold_10-gene-1650.15, Cao1_scaffold_10-gene-1651.6) expanded in the petal of the double flower. Interestingly, these A-class genes had different expression trends. The A-class functional gene (Cao1_scaffold_7-gene-1063.2) was upregulated in the petal and stamen of a double flower, indicating it may be relative to the development of the stamen petaloid. We selected five ABCE class genes for validating the transcriptome data through the RT-qPCR method. Primers were designed by primer5 software (Supplemental Table. S7). Results were highly consistent with the RNA-seq data (Fig. 6), indicating the reliability of our data.

## Discussion

*C. sasanqua* is an important ornamental plant with rich flower architecture variation. Here we sought to understand the transcriptional divergence in floral organs, including comparisons of wild, semi-double, peony double, and rose double flower types. Genes were shared and specified among different double and single flowers were identified to provide targets for flower breeding. In addition, the molecular similarity between the transcriptome of petal and stamen in ZHZR supported the conclusion of stamen petalization.

### The divergence of stamen petaloid influenced double flower architecture

Under the influence of human demand, many double flower cultivars of *C. sasanqua* have been derived, mainly due to the stamen-to-petal transition [11]. This phenomenon has been studied at the molecular level, such as regulated genes of stamen petaloid in *Lagerstroemia speciosa* are identified through performing transcriptome analysis [16]. Transcriptome variation mirror genetic variation [17], we found a significant divergence between wild and double flower and limited divergence between petal and stamen in double flower cultivars. The result is consistent with a previous study, each flower organ is easily separable in wild-type camellia, while petals and stamen gather in double flower cultivars [4]. Over 2000 DEGs were shared by petal and stamen in single and double flower comparisons, respectively. These genes probably significantly contribute to the variation from single to double flowers.

### Phytohormones' response to double flower development

A previous study revealed that plant hormones relate to stamen petaloid [8, 18]. Particularly, the biosynthesis and signal transport of auxin affects the arrangement of the floral whorls [19]. In our results, *CsAUX1* and *CsIAA* in the auxin pathway were upregulated in the petal and stamen of the double flower. The petal primordium is formed by promoting *AUX1* to accumulate auxin, and *PIN-FORMED1* (*PIN1*) transports it [20]. In *Arabidopsis thaliana*, the *IAA1* mutant inhibits the interaction with *TIR1*, resulting in petal loss [21]. Interestingly, *CsSAUR* had a high expression level in sepal, while *AtSAUR* responds to auxin and regulates cell elongation [22], indicating sepal development probably affected double flower variation. Moreover, *CsBSK* in brassinosteroid and *CsARR-B* in the cytokinin pathway probably regulate cell expansion and abnormal flower development, respectively [23, 24]. Genes involved in gibberellin, ethylene, and the abscisic acid pathways also played an important role, suggesting that double flower development is regulated by a complex hormone network.

### Expansin protein probably participates in stamen petaloid

In the coexpression module of *C. sasanqua,* we noted that a model displayed similarity between petal and stamen in double flower, and that cell wall loosening was enriched. The examination of the high weight network, identified some of the expansin proteins, showing an up-regulation in the petal and stamen of the double flower. Petal growth mainly depends on cell expansion [25], and the expansin gene may help wall modification related to petal development [26]. The GA-regulated expansin gene gladiolus (*GgEXPA1*) was expressed prominently in stamen, petal, and tepal expansion [27]. The α-expansins proteins of *Mirabilis jalapa* also show abundant change during the rapid expansion of the ephemeral flowers [28]. Further functional validation is required to elucidate an expansin-mediated mechanism.

### The ABCE model is conservative in the double flower development

In general, the ABCE model defines four regulatory gene functions. A, B, and C class genes work in a combinatorial fashion to confer organ attributes in each whorl [6], and E class genes ensure that all functions are performed normally. We identified homeotic ABCE genes of the MADS family. The A-class genes were upregulated in the stamen and petal of the double flower, indicating double flowers potentially released the constrains of gene expression required for the whorl development. The petal number was increased by heterologous overexpression of *CjAPL2* genes [29]. In contrast, we noted that C class genes were downregulated in the stamen of the double flower. This may be caused by the mutual antagonism between the A-class and C-class function, such that class C activity expands in class A mutant plants [30]. B class genes in *C. sasanqua* have similar expression trends in wild and double flower types, and the result was in agreement with a previous study [4]. Floral organ differentiation required a conserved function of the ABCE gene, but the double flower displayed obscure expression crossing the borders of organ types.

## Conclusion

In short, we found that the designated expression pattern of ABCE genes was deconstructed. Particularly, class A genes activity expands to stamen in double flower. In addition, these genes involved in plant hormone signaling, photosynthesis, and extensin protein were considered candidate regulators of the double flower, but need further investigation to elucidate the complete picture. Our transcriptome database presented here will serve as a useful genetic resource for clarifying double flower domestication.

## Material and method

### Plant materials and RNA extraction

The wild-type *C. sasanqua* (CS) and its cultivated variants (FSZF, ZHZR, XMG) used in the experiment were obtained by the Institute of Subtropical Forestry, Chinese Academy of Forestry (Hangzhou City, Zhejiang Province), and is preserved in the Camellia Germplasm Resource Center (30°05′92′′N, 119°95′94′′E). The deposition number of these samples is as follows: CS: sasanqua, XMG: *Shishigashira*, FSZF: Fuji-no-mine, ZHZR: Shōwa-no-sakae. The formal identification of these *C. sasanqua* cultivars is completed by xinlei Li, zhonglang Wang, and jiyin Gao of the International Camellia Association (camellia.iflora.cn).

The annual rainfall at the study site was 1,500 mm, the soil at the test site was sandy loam, and the pH was 5.5–6.5. Sepal, petal, and stamen of floral organs from wild-type and cultivated camellias were collected, then frozen immediately in liquid nitrogen and stored at − 80 °C. Three biological replicates were obtained from three individuals.

Total RNA of all samples was extracted using the DP441 plant kit (TIAGEN, Beijing, China), following the manufacturer’s instructions, and stored in the freezer before use. Standard-compliant RNA (RIN > 8.0 and concentration > 100 ng/ul) was screened using the NanoDrop1000 (ThermoFisher, Scientific, Wilmington, DE) and Agilent 2100 instruments (Agilent Technologies, Palo Alto, CA, USA).

### Transcriptome sequencing and data processing

According to the manufacturer's instructions, five micrograms of total RNA from each sample were used for constructing the NGS library by mRNA-Seq Sample Prep kit (Illumina Inc., San Diego, CA). Oligo (dT) reads were used to enrich the mRNA, and a fragmentation buffer was used to form short fragments. The short fragments were synthesized into cDNA using DNA polymerase I and RNase. Polymerase chain reaction (PCR) enrichment was performed to obtain the cDNA library [31]. The libraries were sequenced on an Illumina HiSeq 2000 sequencer. The high-quality clean data were mapped to the assembled *C. oleifera* genome data [15]. An index of the reference genome was built using Bowtie v2.2.3, and paired-end clean reads aligned with the reference genome using TopHatv2.0.12. The new genes were predicted via the EMBOSS package (http://emboss.open-bio.org/).

### DEGs identification and functional enrichment analysis

The expression levels of the transcripts were quantified based on the read counts mapped to the genome and were calculated using the Fragments Per Kilobase of transcriptome per Million mapped reads method. DESeq [32] was used for the differential expression analyses between control and experimental groups. The DEGs screening conditions were |log2FC|≥ 1 and false discovery rate < 0.05. The data were compared with the Gene Ontology (GO) databases [33], the Kyoto Encyclopedia of Genes and Genomes [34] (KEGG), and the NR (Non-redundant) protein sequence database [35] (https://ncbi.nlm.nih.gov/blast/db/FASTA/). *P*-value correction was performed using the Benjamini-Hochberg (BH) method, and less than 0.05 were identified as significantly enriched. Moreover, the top GO terms were consolidated into a matrix. R package pheatmap was used for visualization.

### WGCNA and phylogenetic analysis

The R package WGCNA [36] was used to perform coexpression network analysis, and determined the correlation between tissue and module. A positive correlation indicated that the genes of this module had higher expression in this tissue relative to all other samples. Finally, Cytoscape (3.0.0) was used to visualize the network.

At first, the hidden Markov model of MADS and K domain were downloaded in the Pfam [37]. Genes similar to the *CsMADS-box* were searched by the hmm method. Then, these genes were identified by blasting the gene sequences of *Arabidopsis thaliana*. Finally, the results of both parts were combined*.* Sequence alignments were performed using MAFFT [38], and the aligning results were used to build phylogenetic trees by MEGA5.

### Quantitative real-time PCR validation

Primer Premier v5.0 was used to design the gene-specific primers (Supplementary Table S7). The quantitative real-time PCR were performed using the ABI Steponeplus Real-Time PCR System (Thermo, USA) instrument, according to the TB Green Fast qPCR Mix (Takara) instructions. 18 s rRNA gene was used as the internal reference, relative expression level was quantified by the 2^−△△CT^ method [39].

### Statistical analysis

All data were analyzed with three biological replicates. The statistical analysis was conducted using R software. The data are presented as mean ± standard deviation of three biological replicates experiments.

## Supplementary Information

**Additional file 1:** **Table S1.** Summary of sequencing quality.

**Additional file 2:** **Table S2.** The GO enrichment analysis of comparison analysis.

**Additional file 3: Table S3.** The KEGG enrichment analysis of comparison analysis.

**Additional file 4:** **Table S4.** The gene description list of the model.

**Additional file 5: Table S5.** The GO enrichment analysis of coexpression modules.

**Additional file 6: Table S6.** The KEGG enrichment analysis of coexpression modules.

**Additional file 7: Table S7.** The primer of RT-qPCR.

**Additional file 8:** **Figure S1.** The correlation analysis of RNA-seq data.

## Data Availability

The datasets generated and/or analyzed during this study are included in this article, its supplementary information files, or the [NCBI]repository with Accession number: PRJNA837723. [https://www.ncbi.nlm.nih.gov/bioproject/837723].
