# Supplementary figures and images for: Floral organ transcriptome in Camellia sasanqua provided insight into stamen petaloid
Source: BMC Plant Biol. 2022 Oct 5;22:474. doi: 10.1186/s12870-022-03860-x (PMC9535933; doi:10.1186/s12870-022-03860-x)

## Pearson correlation coefficient

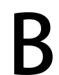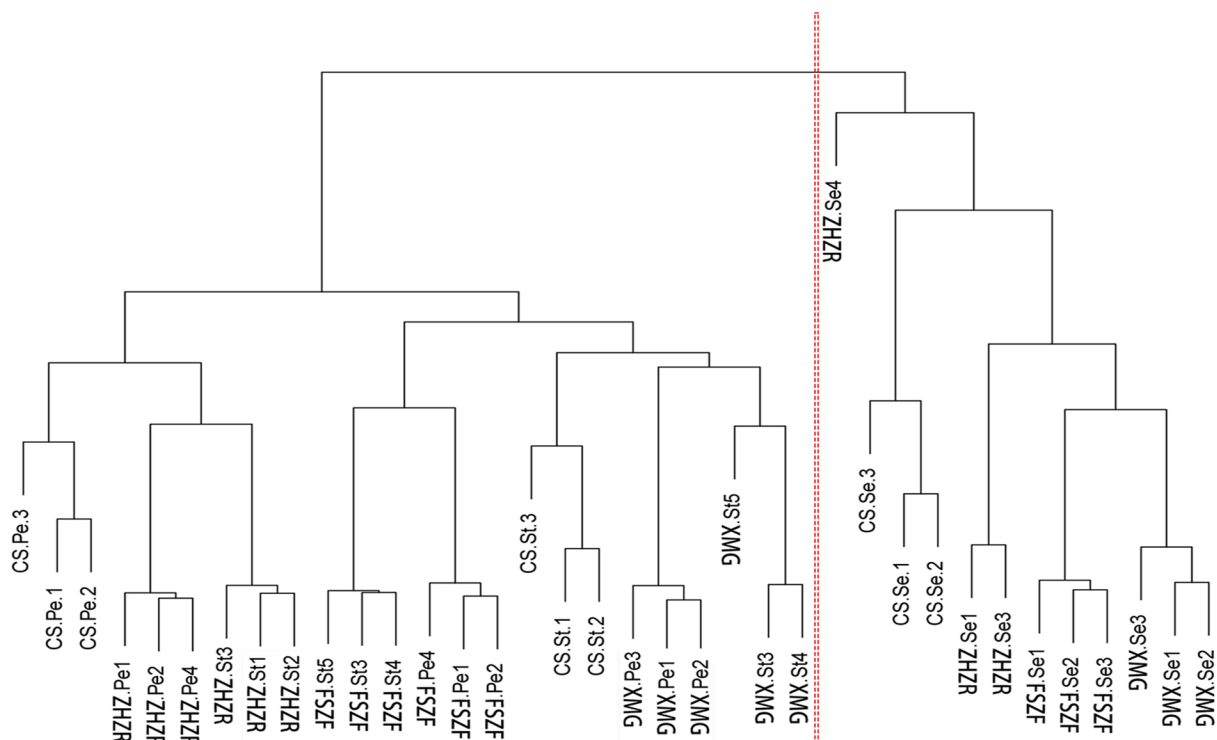

Supplement: Supplementary file 8 — Additional file 8: Figure S1. The correlation analysis of RNA-seq data. [file 12870_2022_3860_MOESM8_ESM.pdf]
